# Constructing a full, multiple-layer interactome for SARS-CoV-2 in the context of lung disease: Linking the virus with human genes and microbes

**DOI:** 10.1371/journal.pcbi.1011222

**Published:** 2023-07-06

**Authors:** Shaoke Lou, Mingjun Yang, Tianxiao Li, Weihao Zhao, Hannah Cevasco, Yucheng T. Yang, Mark Gerstein

**Affiliations:** 1 Program in Computational Biology & Bioinformatics, Yale University, New Haven, Connecticut, United States of America; 2 Department of Molecular Biophysics & Biochemistry, Yale University, New Haven, Connecticut, United States of America; 3 School of Electronic Engineering and Computer Science, Queen Mary University of London, Mile End Road, London, United Kingdom; 4 Institute of Science and Technology for Brain-Inspired Intelligence, Fudan University, Shanghai, China; 5 MOE Key Laboratory of Computational Neuroscience and Brain-Inspired Intelligence, Fudan University, Shanghai, China; 6 MOE Frontiers Center for Brain Science, Fudan University, Shanghai, China; 7 Department of Computer Science, Yale University, New Haven, Connecticut, United States of America; 8 Department of Statistics & Data Science Yale University, New Haven, Connecticut, United States of America; 9 Department of Biomedical Informatics & Data Science, Yale University, New Haven, Connecticut, United States of America; Dartmouth College, UNITED STATES

## Abstract

The COVID-19 pandemic caused by the SARS-CoV-2 virus has resulted in millions of deaths worldwide. The disease presents with various manifestations that can vary in severity and long-term outcomes. Previous efforts have contributed to the development of effective strategies for treatment and prevention by uncovering the mechanism of viral infection. We now know all the direct protein–protein interactions that occur during the lifecycle of SARS-CoV-2 infection, but it is critical to move beyond these known interactions to a comprehensive understanding of the “full interactome” of SARS-CoV-2 infection, which incorporates human microRNAs (miRNAs), additional human protein-coding genes, and exogenous microbes. Potentially, this will help in developing new drugs to treat COVID-19, differentiating the nuances of long COVID, and identifying histopathological signatures in SARS-CoV-2-infected organs. To construct the full interactome, we developed a statistical modeling approach called MLCrosstalk (multiple-layer crosstalk) based on latent Dirichlet allocation. MLCrosstalk integrates data from multiple sources, including microbes, human protein-coding genes, miRNAs, and human protein–protein interactions. It constructs "topics" that group SARS-CoV-2 with genes and microbes based on similar patterns of co-occurrence across patient samples. We use these topics to infer linkages between SARS-CoV-2 and protein-coding genes, miRNAs, and microbes. We then refine these initial linkages using network propagation to contextualize them within a larger framework of network and pathway structures. Using MLCrosstalk, we identified genes in the IL1-processing and VEGFA–VEGFR2 pathways that are linked to SARS-CoV-2. We also found that *Rothia mucilaginosa* and *Prevotella melaninogenica* are positively and negatively correlated with SARS-CoV-2 abundance, a finding corroborated by analysis of single-cell sequencing data.

## Author summary

Our research aimed to understand the full interactome of SARS-CoV-2 infection and develop new treatments for COVID-19. Using a statistical modeling approach called MLCrosstalk, we identified linkages between SARS-CoV-2, human genes, miRNAs, and microbes. Our findings suggest that certain human genes in the IL1-processing and VEGFA–VEGFR2 pathways are linked to SARS-CoV-2, and that the abundance of Rothia mucilaginosa and Prevotella melaninogenica is positively and negatively correlated with SARS-CoV-2 abundance, respectively. Our work offers a unique approach to analyzing the interactions between the virus and various components, with the potential to improve our strategies for treating and preventing COVID-19.

## Introduction

Severe acute respiratory syndrome coronavirus 2 (SARS-CoV-2) has caused one of the deadliest pandemics in human history, infecting more than 600 million people and resulting in more than 6.6 million deaths (WHO, December 2022). While vaccines and antiviral therapies have shown efficacy in reducing the severity of infection, there is still an urgent need to understand the complex interactions between SARS-CoV-2 and human hosts to develop effective methods for diagnosis and treatment, both during infection and its aftermath.

The complete SARS-CoV-2 genome and transcriptome have been studied in-depth [1,2] and combined with mechanistic studies to define the SARS-CoV-2 infection pathway [3,4]. Researchers now have a solid understanding of how SARS-CoV-2 infects cells and which infection-related pathways it activates [5]. This work underpins further analyses on the larger network of interactions and biosignatures in SARS-CoV-2 infection. High-throughput methods have also elucidated interactions between SARS-CoV-2 and the host, shedding light on the host protein/virus protein interaction network [6–8], perturbations in the host gene and cellular networks during the initial stages of SARS-CoV-2 infection (similar to the triggering of cytokine storms) [9], and interactions between host proteins and SARS-CoV-2 RNA during active infection [10]. Single-cell RNA sequencing (scRNA-seq) has provided valuable information regarding biological pathways and biosignatures [11,12] and has revealed the large-scale cellular and molecular landscape of immune responses during SARS-CoV-2 infection in multiple tissues [11,13,14].

Similarly, many independent studies have verified the interaction between microbes and host genes [15], including microRNAs (miRNAs) [16]. Researchers have shown that miRNAs play an important role in antiviral immune responses [17] and participate in the host response to SARS-CoV-2 [18,19], with potential miRNA binding sites in the SARS-CoV-2 genome [19]. Large-scale approaches, as well as computational analyses and modeling with integrated single-cell datasets, have been applied to identify the interactions between host genes and microbes [20,21]. The most general method to identify microbe-associated host genes is to perform a differential expression gene analysis comparing samples with versus without microbes [22]. Some host-responsive genes have been found to associate with certain microbes [23,24]. Correlation analyses can help to further filter microbiota-associated genes from up- and down-regulated differentially expressed genes (DEGs) [23].

Despite these advances in our understanding of SARS-CoV-2 interactions, we lack a holistic model incorporating multiple biological datasets to examine the overall virus–host interaction pattern, with different areas of interest including miRNAs and the microbiome. The examination of the microbiome in the presence of SARS-CoV-2 is one of the most interesting avenues for further study. Previous research has identified the importance of the respiratory microbiome in regulating the immune response to infection [25]. Changes in microbial composition in both the gut and respiratory microbiomes have been observed in COVID-19 patients relative to healthy controls [26–28], with particularly marked decreases in gut bacterial diversity observed in patients with post-acute COVID-19 syndrome (PACS) [29,30]. These findings suggest that microbes play an indispensable role in shaping the host immune response, but their relationship to SARS-CoV-2 infection remains largely unknown. Developing a full interactome will further our knowledge of how SARS-CoV-2 propagates in the body, how it might interact with or alter the prevalence of microbes, and what additional pathways might be activated in PACS. For example, research is currently underway to examine how the reactivation of the Epstein–Barr virus and other pathogens might contribute to PACS [31,32]. These insights warrant further mechanistic study and highlight the need for a comprehensive interactome to explore the relationship between SARS-CoV-2 and the host microbiome.

To date, the challenge in constructing a full interactome has been the integration of multiple layers of information and the identification of inter-layer associations relevant to the host response in SARS-CoV-2 infection. We developed MLCrosstalk to overcome these challenges for defining host–pathogen interactions. MLCrosstalk incorporates multiple data sources and data types (e.g., miRNA, microbes, protein-coding genes, and protein–protein interactions) to identify COVID-19-specific host gene–microbiome interactomes in different tissues across patient samples, which we term the “full interactome”. With network propagation analysis, we further refined the interactome based on signaling pathways. For this paper, we applied MLCrosstalk to achieve two main objectives: 1) to identify interaction patterns between SARS-CoV-2 and microbes and 2) to discover microbe-linked gene pathways differentially activated in COVID-19 patients compared with community acquired pneumonia (CAP) patients and healthy individuals.

## Results

### MLCrosstalk model

We briefly describe MLCrosstalk here for clarity, but a detailed description can be found in the Methods section.

**Fig 1:**
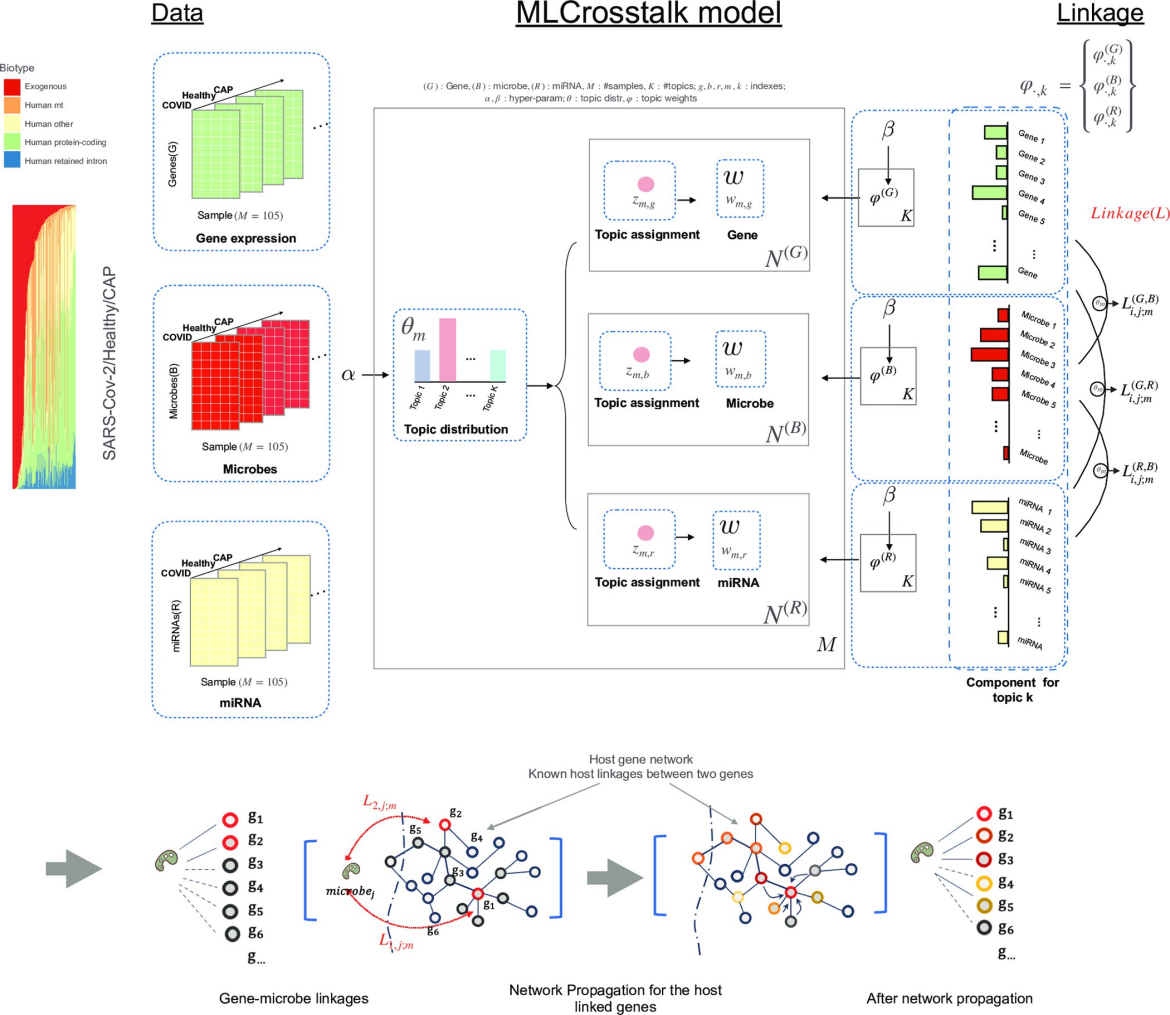
MLCrosstalk workflow. Data include gene expression, microbe abundance, and (pre)miRNA expression matrices. These data are then inputted into MLCrosstalk to infer linkages. After modeling, we apply network propagation to refine the linkages.

The input of MLCrosstalk is a matrix of 105 patient samples with dimensions describing features such as gene and miRNA expression and microbe abundance (shown in Fig 1). Although superficial correlations across patients can be identified between two parts of the matrix such as gene and microbes (i.e., rows in Fig 1), the overall dataset is too noisy to produce meaningful results. To address this, MLCrosstalk uses the latent Dirichlet allocation procedure to create topics that group genes, microbes, and miRNAs with similar co-occurrence patterns across patient samples. The resulting topic matrix (*φ* in Fig 1 with *k* topics) is similar to the derivation of gene expression signatures in non-negative matrix factorization. Within each patient, the weights for each topic are specified by a vector *θ* (in Fig 1). For a given gene *i* and microbe *j*, we can determine the level of correlation of their representation across the various topics to obtain a raw linkage score. This score can be further normalized by comparison to a background distribution of all possible scores and then individualized to a particular patient *m* by considering only the relevant topics active in that patient (the final score is indicated by *L*_*i*,*j*:*m*_ in Fig 1 and represents a statistical significance value from the distribution). From this, we can link a particular microbe to a human gene or miRNA. These linkages are further refined and related to known pathways using network propagation (also shown in Fig 1) to obtain a final set of linkages.

MLCrosstalk has four key advantages for integrating multiple data types. First, it takes advantage of the Dirichlet distribution of hyperparameters to handle sparse and noisy data. Second, it enforces a unitary topic distribution for each sample, allowing for easy comparison across samples and facilitating linkage identification between different data types. Third, it can be easily extended to multiple data types. Fourth, it can infer specific individual linkages. Fig 1 shows the MLCrosstalk workflow.

**Fig 2:**
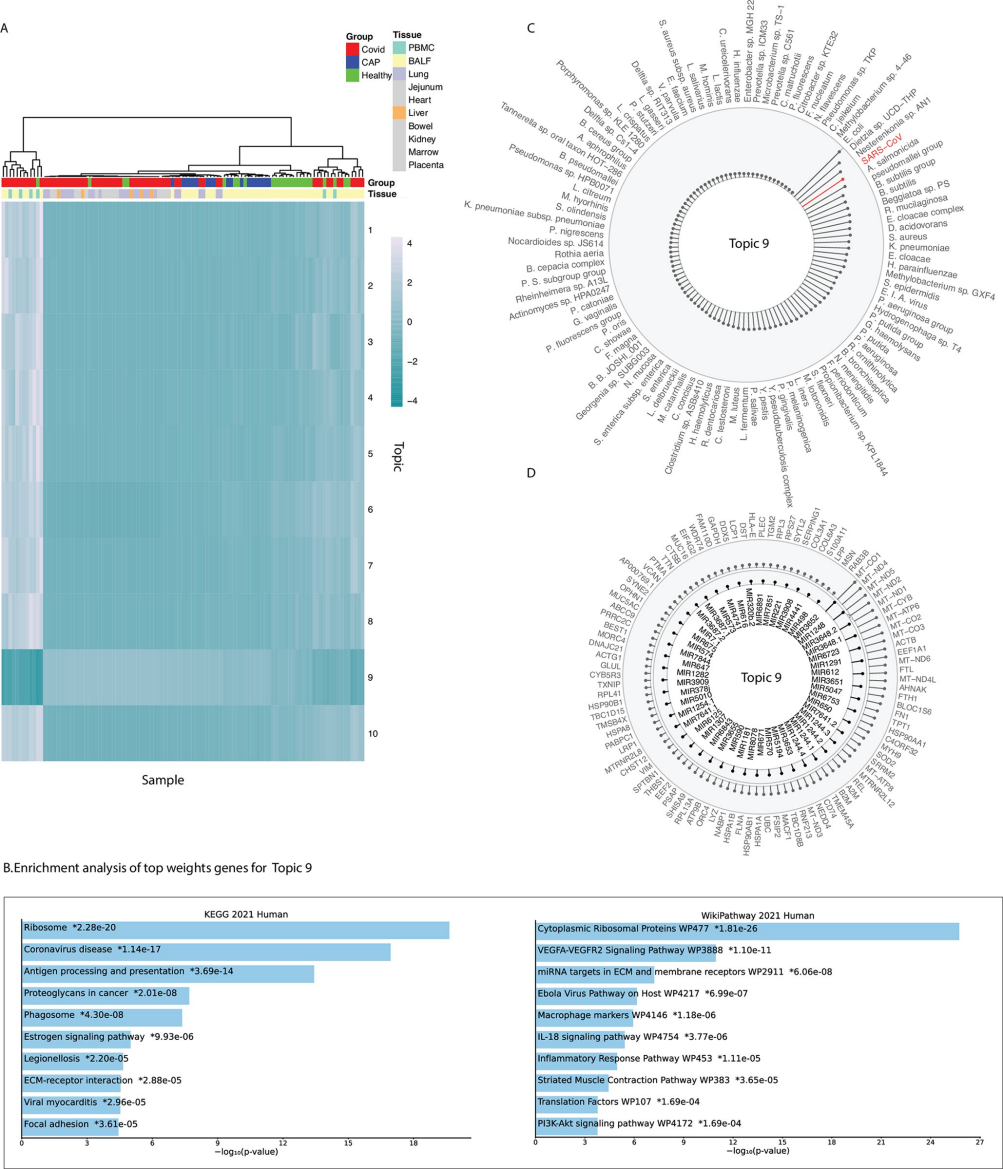
Model evaluation and functional analysis. (A) Heatmap of the topic distribution across all 105 samples. (B) Functional enrichment analysis of topic 9. (C–D) Top-weighted protein-coding genes, pre-miRNAs, and microbes for topic 9.

In our study of COVID-19 datasets (see data sources in S1 Fig), we applied MLCrosstalk to extract dimensionally reduced patterns (topics) from the data matrix to infer comprehensive linkages among host protein-coding genes, noncoding genes (e.g., miRNA), and microbes. Based on the topic distribution matrix, distinct clusters emerged for COVID-19 patients, community acquired pneumonia (CAP) patients, and healthy individuals (Fig 2A). By comparing topic distribution to a random background, we identified topic 9 as the most biologically interesting cluster, with top-weighted genes enriched in immune-related and SARS-CoV-2-related pathways (Figs 2B, S2 and S3; for additional topics, see S1 Table) based on a similar approach as in our previous work [33]. Fig 2C and 2D displays the top-weighted protein-coding genes, miRNAs, and microbes associated with topic 9, with SARS-CoV-2 being one of the strongest microbe contributors.

### SARS-CoV-2 links to microbes

**Fig 3:**
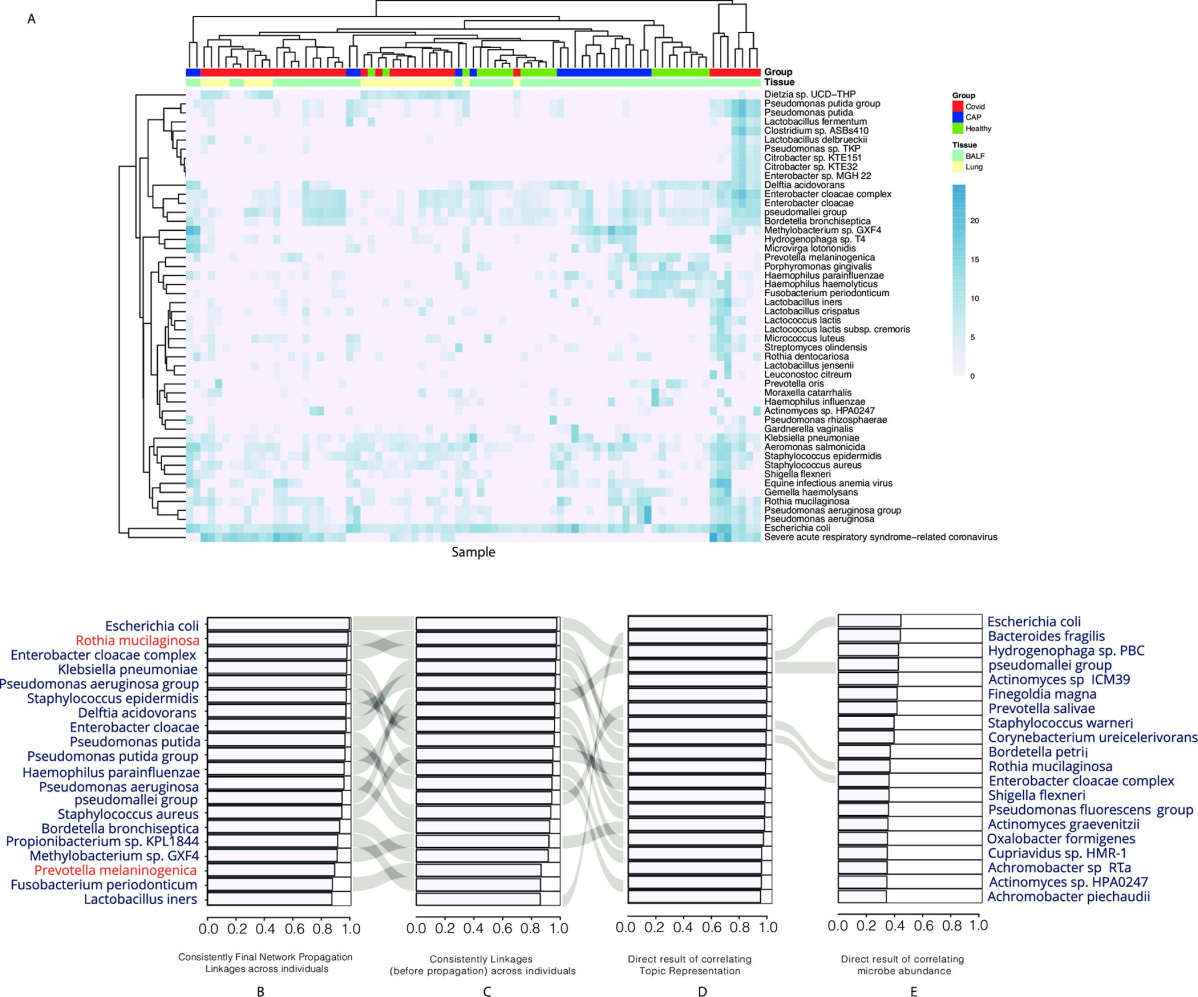
SARS-CoV-2 and microbe association analysis. (A) Heatmap of the most abundant microbes, including SARS-CoV-2. (B-E) Microbes associated with COVID-19 using a (B) two-step linkage-based approach with network propagation, (C) two-step linkage-based approach without network propagation, (D) correlation based on latent microbe topic components, and (E) correlation based on abundance.

SARS-CoV-2 was one of the most detected microbes in the COVID-19 patient samples (Fig 3A). We associated the top 100 most abundant microbes with SARS-CoV-2 by comparing the final network propagation linkages across individuals (Fig 3B). The progression in Fig 3C–3E shows how linkages overlap using different methods, where Fig 3C shows the top SARS-CoV-2-associated microbes based on consistent linkages (before propagation) across individuals, Fig 3D shows the direct result of correlating topic representations for each microbe with SARS-CoV-2, and Fig 3E shows the direct result of correlating microbe abundance.

According to the analysis (Fig 3B), SARS-CoV-2 is linked to the abundance of several well-known pathogens, including *Rothia mucilaginosa*, *Fusobacterium periodonticum*, *Prevotella melaninogenica*, and *Haemophilus parainfluenzae* [34, 35]. Although other microbes such as *Escherichia coli*, *Enterobacter cloacae complex*, *Klebsiella pneumoniae*, *Pseudomonas aeruginosa*, and *Staphylococcus aureus* are highly associated with COVID-19, they are commonly found as hospital-acquired species and are therefore not the focus of our analysis [36,37].

### SARS-CoV-2-associated microbes show distinct patterns

**Fig 4:**
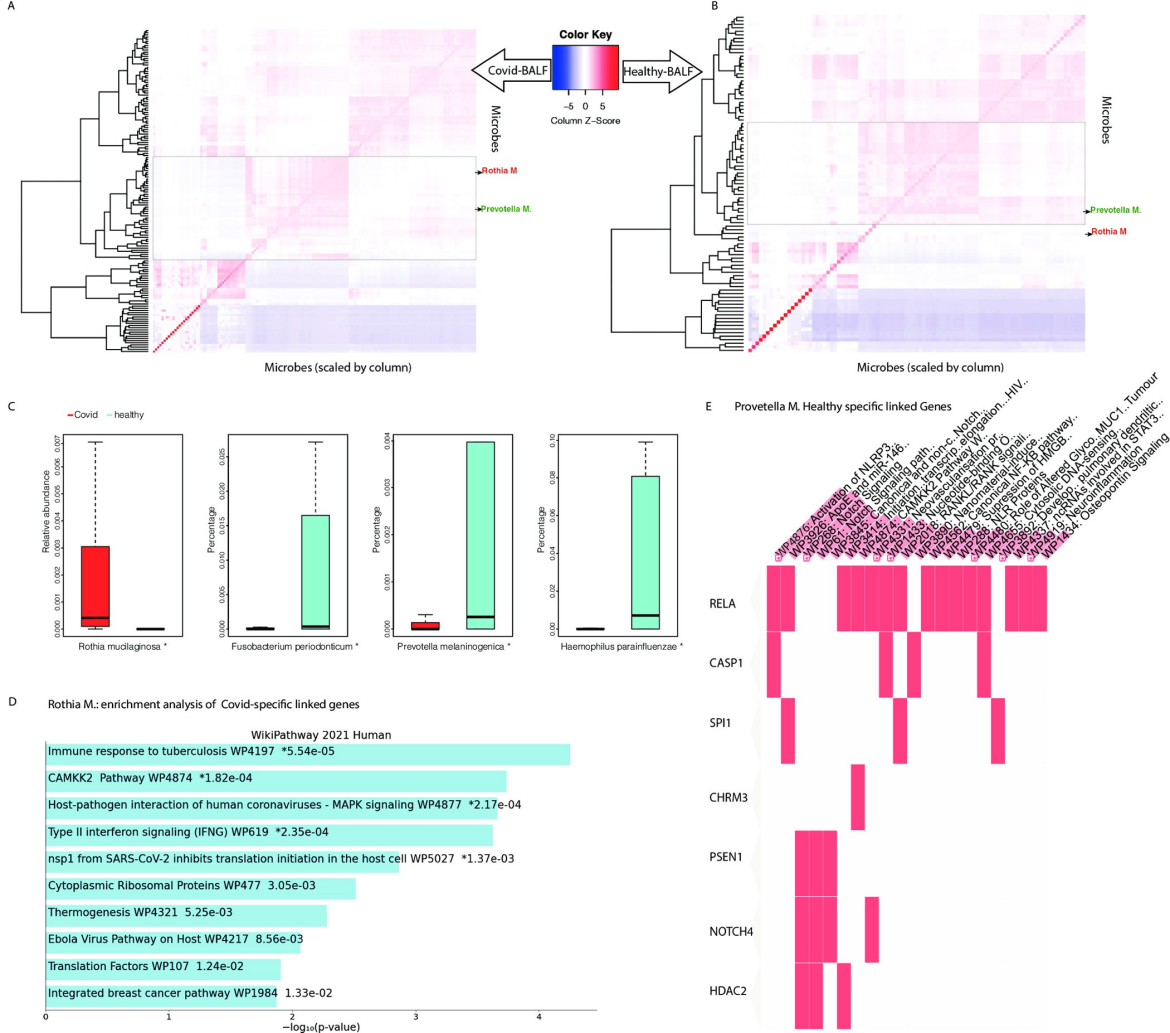
Microbe association patterns with SARS-CoV-2. (A) Heatmap of microbe clusters in the COVID-19 patient group. (B) Heatmap of microbe clusters in healthy patients. (C) Abundance of top SARS-CoV-2-associated microbes between COVID-19 and healthy patients. (D) Functional enrichment of *R*. *mucilaginosa*-linked, COVID-19-specific genes. (E) Functional enrichment of *P*. *melaninogenica*-linked healthy-specific genes.

Microbes found to co-occur with SARS-CoV-2 exhibited varying interaction patterns in bronchoalveolar lavage fluid (BALF) between 19 COVID-19 patients and 18 healthy individuals (Figs 4A, 4B and S4). In BALF, we observed significant changes in microbe abundance, including an increased abundance of *R*. *mucilaginosa* and a decreased abundance of *P*. *melaninogenica* (Fig 4C), which is linked with different sets of miRNAs (S4 Fig). These findings suggest that the microbes may have distinct roles in response to SARS-CoV-2 infection.

*R*. *mucilaginosa*, which is a gram-positive coccus found in the oropharynx and upper respiratory tract, plays an anti-inflammatory role in the respiratory microbiome [38,39]. The significantly high abundance of *R*. *mucilaginosa* in COVID-19 patients (Fig 4C) led us to study specific linked genes in COVID-19 versus healthy groups. This analysis revealed that the enriched gene sets for COVID-19 are more related to immune response, host-pathogen interaction, and SARS-CoV-2-associated genes (Fig 4D).

In contrast to *R*. *mucilaginosa*, *F*. *periodonticum*, *P*. *melaninogenica*, and *H*. *parainfluenzae* exhibited significantly reduced relative abundance in COVID-19 patients. Research has shown that *P*. *melaninogenica* and *H*. *influenzae* can induce general respiratory inflammation accompanied by lung neutrophilia [40]. We found that *P*. *melaninogenica-*linked genes are enriched in NLRP3 activation and NF-κβ pathways in healthy individuals (Fig 4E; see detailed pathway information in S2 Table), suggesting that these microbes in the respiratory microbiome may cause modest inflammatory effects that can be controlled by the host. However, in COVID-19 patients, the stronger inflammatory response triggered by SARS-CoV-2 may require a more drastic host immune response that includes the suppression or removal of these inflammatory microbes, leading to their decreased abundance.

### SARS-CoV-2 associations with tissues, genes, and pathways

**Fig 5:**
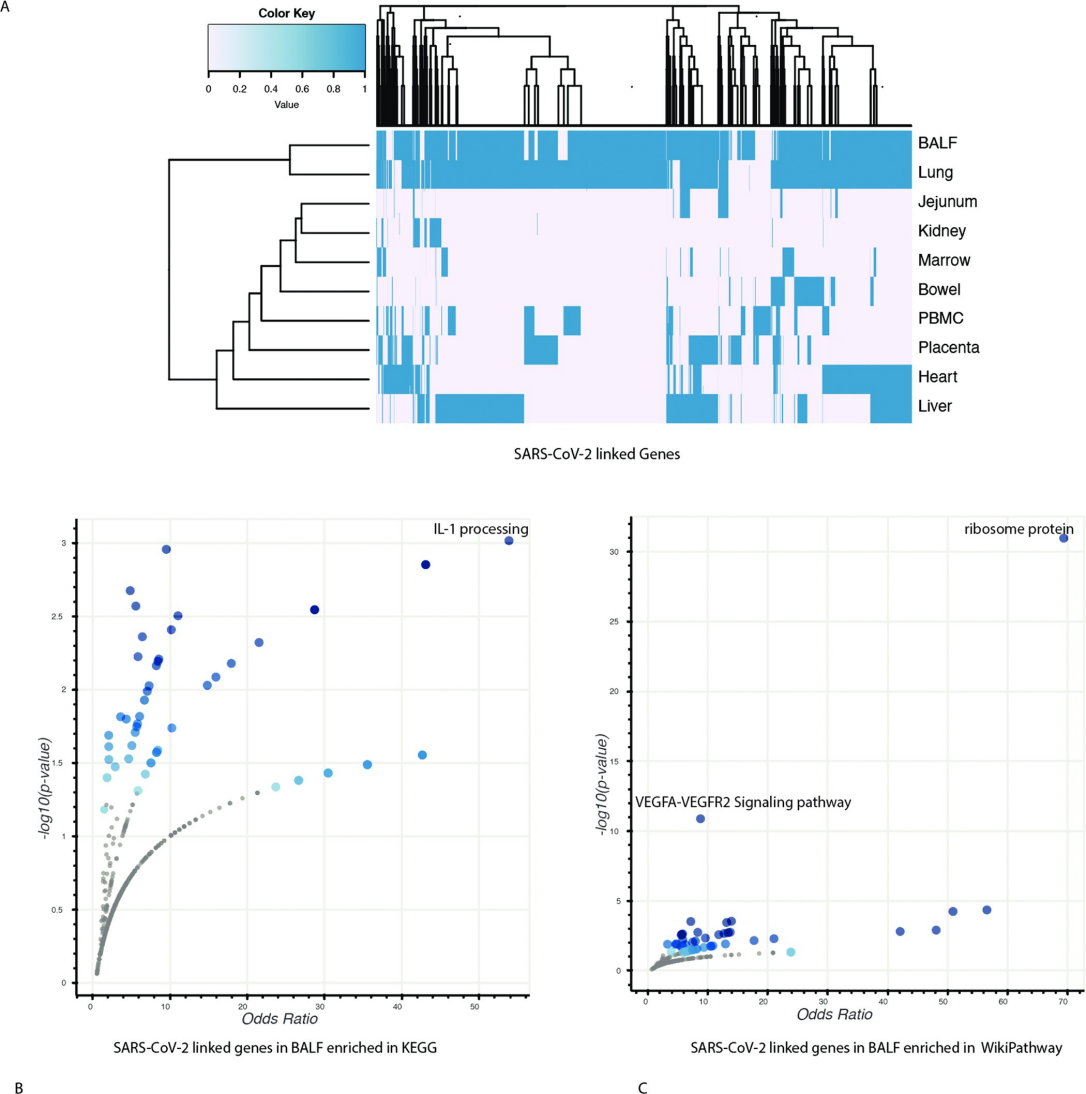
SARS-CoV-2-linked genes in multiple tissues. (A) Cluster of genes associated with SARS-CoV-2 in multiple tissues. (B) Enrichment analysis of SARS-CoV-2-associated genes in KEGG. (C) Enrichment analysis of SARS-CoV-2-associated genes in Wikipathway.

The linkages among host protein-coding genes, miRNAs, and microbes can lead to extensive changes and connections following SARS-CoV-2 infection. To investigate these linkages, we examined gene–microbe and miRNA–microbe connections in 10 different tissues and sample types from COVID-19 patients, including BALF, bowel, heart, jejunum, kidney, liver, lung, marrow, peripheral blood mononuclear cells, and placenta. The resulting clusters of genes, microbes, and miRNAs displayed tissue-specific patterns, particularly for BALF and lung tissue (Figs 5A and S5; Also, S4 Table connects these genes to known human variants from HGI).

We selected BALF to compare linkages across COVID-19, CAP, and healthy samples, as it was the only sample type with available data for all three groups. Our analysis showed that the COVID-19 group had more microbe-linked genes than the healthy group. Further, the genes associated with SARS-CoV-2 were significantly enriched in the IL1 processing pathway (Fig 5B) and in the VEGFA–VEGFR2 pathway (Fig 5C; the ribosome protein gene set was not included due to potential experimental bias), highlighting the importance of the immune response and viral entry in the SARS-CoV-2 and host interaction.

### Cell-type-specific effect on host response

**Fig 6:**
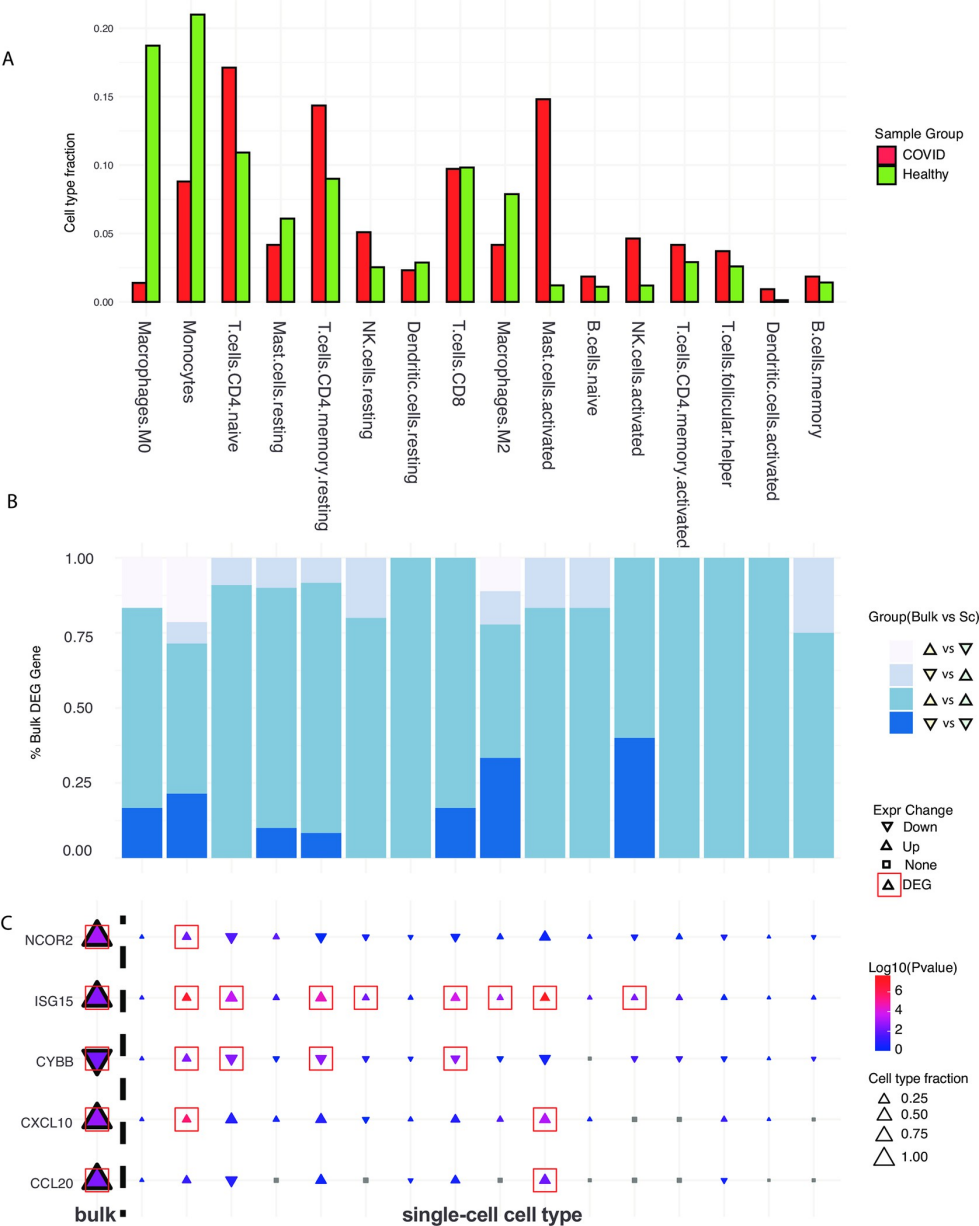
Cell-type-specific host response. A) Cell-type fraction changes between the COVID-19 and healthy group. B) Gene expression changes between bulk cell and single-cell for the COVID-19 versus healthy groups. C) *R*. *mucilaginosa*-linked DEGs show cell-type-specific changes.

We utilized scRNA-seq data to investigate cell-type-specific responses of DEGs associated with *R*. *mucilaginosa* in healthy and SARS-CoV-2-infected cells. Our analysis revealed that the major cell types in healthy samples were monocytes, M0 macrophages, and naïve T cells, whereas in SARS-CoV-2-infected samples, the major cell types were Mast and T cells, which are involved in active immune responses (Fig 6A).

We observed good consistency in gene expression changes between bulk RNA-seq and scRNA-seq data, with over 50% of genes showing the same trend of up- or down-regulation in all cell types. Moreover, considering only the DEGs (p-value < 0.05) from bulk RNA-seq, the ratio of consistent genes from the scRNA-seq analysis increased up to 100% for some cell types such as memory-activated CD4 T cells, activated dendritic cells, and CD8 T cells (Fig 6B). Additionally, the DEGs from *R*. *mucilaginosa*-linked genes in type II interferon signaling and SARS-CoV-2-related pathways, showed significant up/down-regulation in both bulk RNA-seq and scRNA-seq analysis. Notably, NCOR2 [41], ISG15 [42], CYBB [43], CXCL10 [41,44], and CCL20 [45], which are all known to be associated with SARS-CoV-2 infection, exhibited significant expression changes (Fig 6C). Monocytes showed significantly high expression of NCOR2, ISG15, CYBB, and CXCL10. T cells exhibited high expression of ISG15, which has been reported to exacerbate inflammation during COVID-19 infection [46]. In addition, T cells showed significantly down-regulated CYBB, which potentially triggers a higher immune response, and up-regulation of genes enriched in the IL-18, NF-κβ, and type-1 interferon pathways [43]. These results provide evidence of cell-type-specific effects after SARS-CoV-2 infection.

## Discussion

We developed MLCrosstalk to address three major challenges in integrative data mining: noisy and heterogeneous data, unitary topic distribution, multiple-type data integration, and personalized linkage identification. Using the SARS-CoV-2 dataset as an example, we demonstrate that MLCrosstalk can capture latent patterns in multiple data types and infer sample-specific linkages that are supported by biological evidence.

MLCrosstalk extends latent Dirichlet allocation and handles noisy and missing data by enforcing a unified topic distribution. By doing so, MLCrosstalk controls the sparsity of topics and components and builds a latent representation of multiple data types within the same topic. Unlike alternative methods that can infer overall associations using large cohorts, MLCrosstalk infers sample-based linkages by considering the effect of topic distribution in each sample.

The COVID-19 pandemic is a critical public health crisis that demands a deeper understanding of the underlying biology to develop effective treatment strategies. Our MLCrosstalk method can integrate various data types and uncover hidden patterns without supervision. Through MLCrosstalk, we identified linkages between genes and microbes and refined the identifications by integrating biological pathways via network propagation. Our findings show distinct patterns of microbes in COVID-19 patients, such as a significantly increased and decreased relative abundance of *R*. *mucilaginosa* and *P*. *melaninogenica*, respectively. Additionally, we discovered genes associated with SARS-CoV-2 and *R*. *mucilaginosa* and identified gene-enriched pathways, including the VEGFA–VEGFR2, type II interferon, and SARS-CoV-2 signaling pathways. Furthermore, our study integrated scRNA-seq data to reveal that the host response to microbes is cell-type specific.

## Methods

### Data collection and processing

This study included 105 data samples from two studies by Desai and Shen and colleagues [47,48]. See S1 Fig for details on the data sources. The dataset from Desai et al. includes COVID-19 samples from multiple tissues, whereas the Shen et al. dataset includes COVID-19, CAP, and healthy samples for comparison of lung function changes. These large-scale datasets from multiple tissues enabled us to compare the different interactomes between lung disease and SARS-CoV-2, as well as host responses in different tissues after SARS-CoV-2 infection. The transcriptome data were analyzed using the exceRpt pipeline. Briefly, RNA-seq reads were subjected to quality assessment using FastQC software v.0.10.1 both prior to and following 3′ adapter clipping. Adapters were removed using FastX v.0.0.13. Identical reads were counted and collapsed to a single entry and reads containing Ns were removed. Clipped and collapsed reads were filtered through the Univec database of common laboratory contaminants and a human ribosomal database before mapping to the human reference genome (hg19) and pre-miRNA sequences using STAR [49]. Reads that did not align were mapped against a ribosomal reference library of bacteria, fungi, and archaea, compiled by the Ribosome Database Project [50], and then mapped to genomes of bacteria, fungi, plants, and viruses, retrieved from GenBank [51]. In cases where RNA-seq reads aligned equally well to more than one microbe, a “last common ancestor” approach was used, and the read was assigned to the next node up the phylogenetic tree, as performed by similar algorithms [52,53].

Gene expression, pre-miRNA and exogenous genomic, and rRNA frequency were generated by exceRpt [52,53]. Exogenous content was filtered to remove the potential contaminants and to keep only pathogenic microbes. The gene expression values of COVID-19, CAP, and healthy individuals were quantile normalized and converted to integers with microbe and miRNA frequency.

### MLCrosstalk model

As shown in Fig 1, we extended a topic modeling algorithm that can integrate multiple data types. To make the continuous data work on the topic model, all of the continuous values were converted into integers and scaled down to reduce computational intensity.

For any patient group or sample, *M* denotes the number of individuals or samples (here it is 105), which is indexed by *m*; *K* is the number of topics (here it is 10), which is indexed by *k*; *θ* represents the document to topic distribution, or topics; *φ* denotes the word-to-topic distribution, or topic component; and *α*, *β* are the hyper-parameters of the document-to-topic distribution. The input matrices include gene, microbe, and (pre)-miRNA abundances, for which each row represents a corresponding sample, and each column is a gene, microbe, or miRNA, respectively.

In the MLCrosstalk model, the superscript (*G*), (*R*), and (*B*) represent gene, (pre)-miRNA, and microbe data types, respectively, and *g*, *r*, and *b* are the index; *N*^(*G*)^, *N*^(*R*)^*N*^(*B*)^ is the total number of words (genes, miRNAs, or microbes); *Z*, *W* (*or z*, *w*) are the assigned topic and word, respectively. The joint distribution *P*(*Z*, *W*; *α*, *β*) can be derived as:

$$P(Z,W;\;\alpha ,\beta )=P(Z^{\left(G\right)},W^{\left(G\right)},Z^{\left(R\right)},W^{\left(R\right)},Z^{\left(B\right)},W^{\left(B\right)};\alpha ,\beta )=\int_{\theta }\int_{\varphi^{\left(g\right)}}\int_{\varphi^{\left(r\right)}}\int_{\varphi^{\left(b\right)}}{P(Z}^{\left(G\right)},W^{\left(G\right)},{\varphi^{\left(G\right)},Z}^{\left(R\right)},W^{\left(R\right)},\varphi^{\left(R\right)},Z^{\left(B\right)},W^{\left(B\right)},\varphi^{\left(B\right)},\theta ,\alpha ,\beta )d\theta d\varphi^{\left(G\right)}d\varphi^{\left(R\right)}d\varphi^{\left(B\right)}$$

$$=\int \prod_{m=1}^{M}P(\theta_{m};\alpha )\prod_{g=1}^{N^{\left(G\right)}}p(Z_{m,g}^{\left(G\right)}|\theta )\prod_{r=1}^{N^{\left(R\right)}}p(Z_{m,r}^{\left(R\right)}|\theta )\prod_{b=1}^{N^{\left(B\right)}}p(Z_{m,b}^{\left(B\right)}|\theta )d\theta \times \int \prod_{k=1}^{K}P(\varphi_{k}^{\left(G\right)};\beta )\prod_{m=1}^{M}\prod_{g=1}^{N^{\left(G\right)}}p(W_{m,g}^{\left(G\right)}|\varphi_{Z_{m,g}}^{\left(G\right)})d\varphi^{\left(G\right)}\times \int \prod_{k=1}^{K}P(\varphi_{k}^{\left(R\right)};\beta )\prod_{m=1}^{M}\prod_{r=1}^{N^{\left(R\right)}}p(W_{m,r}^{\left(R\right)}\varphi_{Z_{m,r}}^{\left(R\right)})d\varphi^{\left(R\right)}\times \int \prod_{k=1}^{K}P(\varphi_{k}^{\left(B\right)};\beta )\prod_{m=1}^{M}\prod_{b=1}^{N^{\left(B\right)}}p(W_{m,b}^{\left(B\right)}|\varphi_{Z_{m,b}}^{\left(B\right)})d\varphi^{\left(B\right)}$$

$$=\prod_{m=1}^{M}\frac{\Delta (n_{m,(.),(.)}^{\left(G\right)}+n_{m,(.),(.)}^{\left(R\right)}+n_{m,(.),(.)}^{\left(B\right)}+\alpha )}{\Delta (\alpha )}\prod_{k=1}^{K}\frac{\Delta (n_{(.),(.),k}^{\left(G\right)}+\beta )}{\Delta (\beta )}\prod_{k=1}^{K}\frac{\Delta (n_{(.),(.),k}^{\left(R\right)}+\beta )}{\Delta (\beta )}\prod_{k=1}^{K}\frac{\Delta (n_{(.),(.),k}^{\left(B\right)}+\beta )}{\Delta (\beta )}$$

*I*^*G*^,*I*^*B*^,*I*^*R*^ is the matrix indicator for expression and abundance, where $I=\left\{ \begin{array}{cc} 1 & if\;expr\;or\;abundance>0\\ 0 & if\;expr\;or\;abundance>0 \end{array}\right.$ and *I* is the matrix of #word(gene, microbe or miRNA) by #sample (m).

The raw link score *l*_*i*,*j*_ can be defined as

$l_{i,j;x,y,m}=l(x_{i}∼y_{j}|m)=\frac{\sum_{k=1}^{K}{I_{x,m}I_{y,m}\theta }_{m,k}\varphi_{i,k}^{\left(x\right)}\varphi_{j,k}^{\left(y\right)}}{\left\|\varphi_{i,∙}^{\left(x\right)}\|\|\varphi_{j,∙}^{\left(y\right)}\right\|}$, where *x*, *y* represent gene(G), microbe(B), and miRNA (R). For example, $\varphi_{i,k}^{\left(g\right)}$ is the topic component of gene *i*, $\varphi_{j,k}^{\left(b\right)}$ is the topic component of microbe *j*, and the raw link score *l*_*i*,*j*;*m*_

$$=(\sum_{k=1}^{K}{I_{m}I_{y,m}\theta }_{m,k}\varphi_{i,k}^{\left(g\right)}\varphi_{j,k}^{\left(b\right)})/(\|\varphi_{i,k}^{\left(g\right)}\|\|\varphi_{i,k}^{\left(b\right)}\|)$$

To infer a background *l*′ of *l*_*i*,*j; x*,*y*,*m*_, we shuffle the *φ*^(*g*)^, *φ*^(*b*)^ for each topic *k* and then calculate the ${l^{\prime }}_{a,b}^{m}$ for 1,000 times and use the mean and variance to infer the one-tailed *p*-value. We then use the false discovery rate adjustment to get a *q*-value for the inference of linkages *L* for each sample.

### Pathway integration and curation

We used the Pathway Commons v12 all-database version as a base, and then integrated the latest online version of KEGG (July 16, 2021) and Reactome (July 3, 2021) to output all the gene pair lists. We also combined the pathway information from WikiPathways (May 10, 2021) and gene symbols from the HUGO Gene Nomenclature Committee with the gene pair list. Finally, we obtained the gene pair list with pathway information.

### Network propagation

We generated a gene–gene interaction map based on the latest version of several protein–protein interaction databases (KEGG, Reactome, and WikiPathways), in which each node represents a gene, or a protein and each edge represents a gene–gene connection or protein–protein interaction. Then, we applied the Random Walk with Restart (RWR) algorithm on the network using the *q*-value (*L*._,*j*_ denoted by *q*_*j*_) of the microbe (*j*)–gene linkage restart as the node value. *W* is adjacent matrix, and *r* is an arbitrary value (0.3).


$$q_{j}^{t+1}=(1-r)Wq_{j}^{t}+rq_{j}^{0}$$

After RWR convergence, we identified the top-ranked significant linked genes based on the final propagated value *q*. Everything above the significance threshold is now linked back to microbe, resulting in our final gene-to-microbe linkages after propagation.

### Single-cell RNA-seq data analysis

We downloaded scRNA-seq BALF data from healthy controls and COVID-19 patients from a publicly available resource (GSE145926). The scRNA-seq data were processed and mapped to a mixed genome (human hg38 + SARS-CoV-2 ASM985889v3) by CellRanger (v7.0.1) with default parameters. Then, we obtained dense expression matrices by the CellRanger mat2csv utility function.

COVID-associated cells were identified by at least one COVID gene count in each barcode. Cell types of the scRNA-seq data were assigned by CIBERSORT with the LM22 signature matrix. The cell type of each barcode was determined by the cell type in LM22 that had the highest composition.

For gene significance comparison, all COVID cells in the three COVID patient samples (SRR11537949, SRR11537950, SRR11537951) were used for analysis (S3 Table). For healthy cells, we selected a corresponding number of healthy cells from SRR11537948 and ensured an equal number of COVID cells and healthy cells for each cell type for comparison. We compared the log2CPM of each gene in the pathway with the Wilcoxon test.

## Supporting information

S1 Fig Summary of samples. Left, middle, and right pie charts represent composition of samples under different categories: BALF tissue, tissues in healthy individuals, and tissues in COVID-19 patients. The number in the brackets represents the number of samples for that group.
(TIF)

S2 Fig Enrichment analysis for topic 9 in the virus–host protein–protein interactions gene sets. Enrichment analysis of the top 100 genes of topic 9 in the virus–host protein–protein interactions gene sets.
(TIF)

S3 Fig Enrichment analysis for topic 9 in the COVID-19-related gene sets. Enrichment analysis of the top 100 genes of topic 9 in the COVID-19-related gene sets.
(TIF)

S4 Fig **Microbe clusters in (A) COVID-19 and (B) healthy BALF tissue samples.** The SARS-CoV-2-associated microbes are labeled. (C) *R*. *mucilaginosa*- and (D) *P*. *melaninogenica*-associated miRNA in COVID-19 patients. The miRNA in the pink circle is a known miRNA associated with SARS-CoV-2.
(TIF)

S5 Fig Heatmap of (A) miRNA and (B) microbe linkages across multiple tissues.
(TIF)

S1 Table Biological annotation of topics based on the enrichment analysis of top-weighted genes. (XLSX)

S2 Table Enrichment analysis results for clustergram in Fig 4E. (XLSX)

S3 Table Summary of scRNA-seq data analysis. (XLSX)

S4 Table Overlap between Host Genetics Initiative (HGI) associated genes with SARS-CoV-2 linked gene lists inferred by MLCrosstalk. (XLSX)
