# Supplementary figures and images for: Constructing a full, multiple-layer interactome for SARS-CoV-2 in the context of lung disease: Linking the virus with human genes and microbes
Source: PLoS Comput Biol. 2023 Jul 6;19(7):e1011222. doi: 10.1371/journal.pcbi.1011222 (PMC10325097; doi:10.1371/journal.pcbi.1011222)

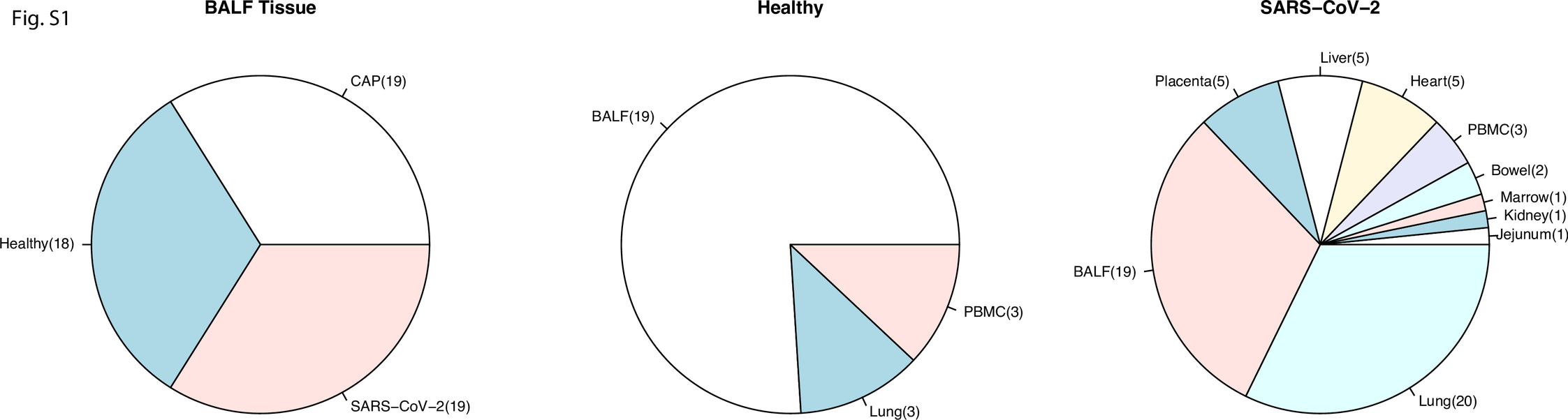

Supplement: S1 Fig — Left, middle, and right pie charts represent composition of samples under different categories: BALF tissue, tissues in healthy individuals, and tissues in COVID-19 patients. The number in the brackets represents the number of samples for that group. (TIF) [file pcbi.1011222.s001.tif]

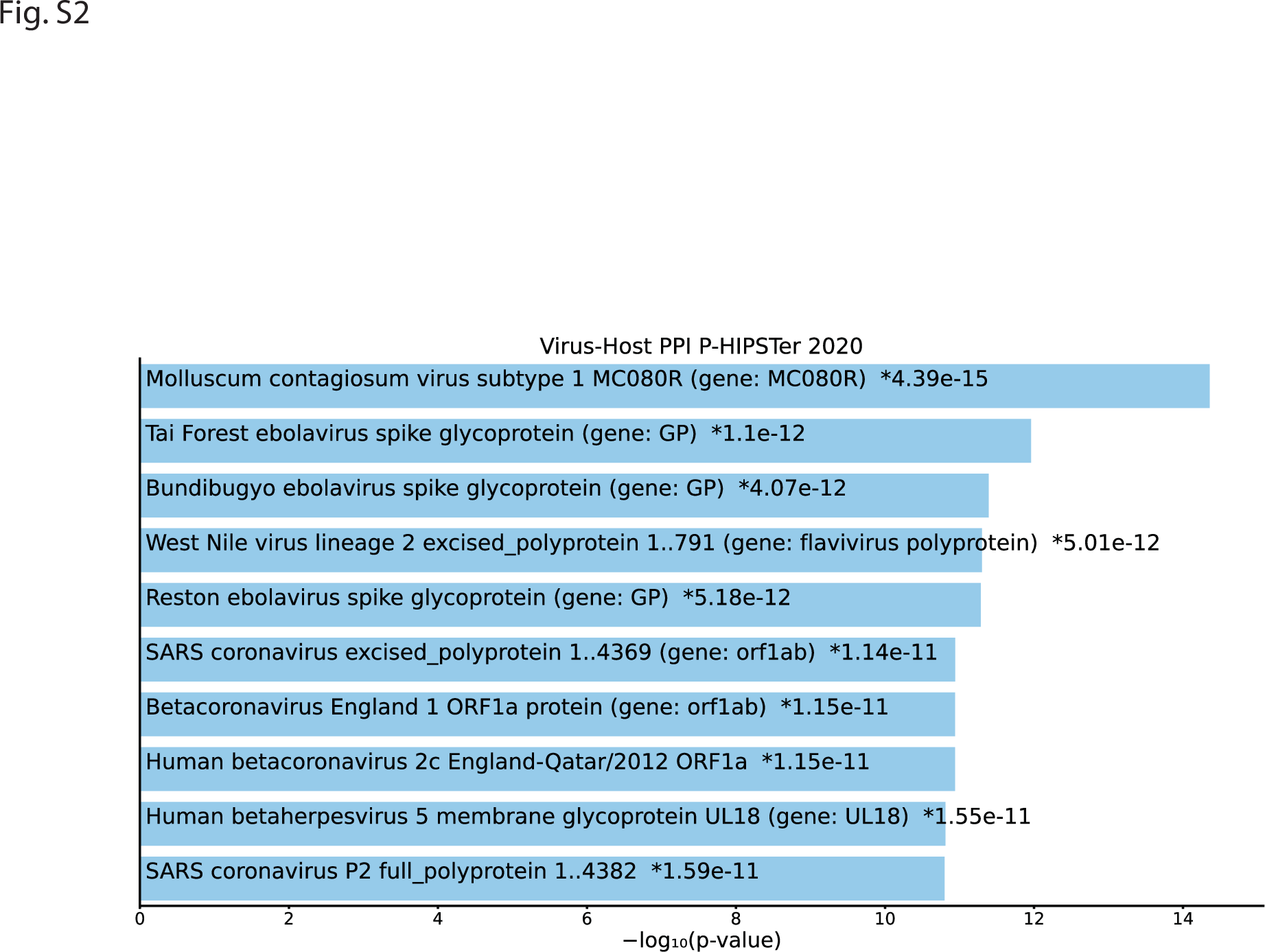

Supplement: S2 Fig — Enrichment analysis of the top 100 genes of topic 9 in the virus–host protein–protein interactions gene sets. (TIF) [file pcbi.1011222.s002.tif]

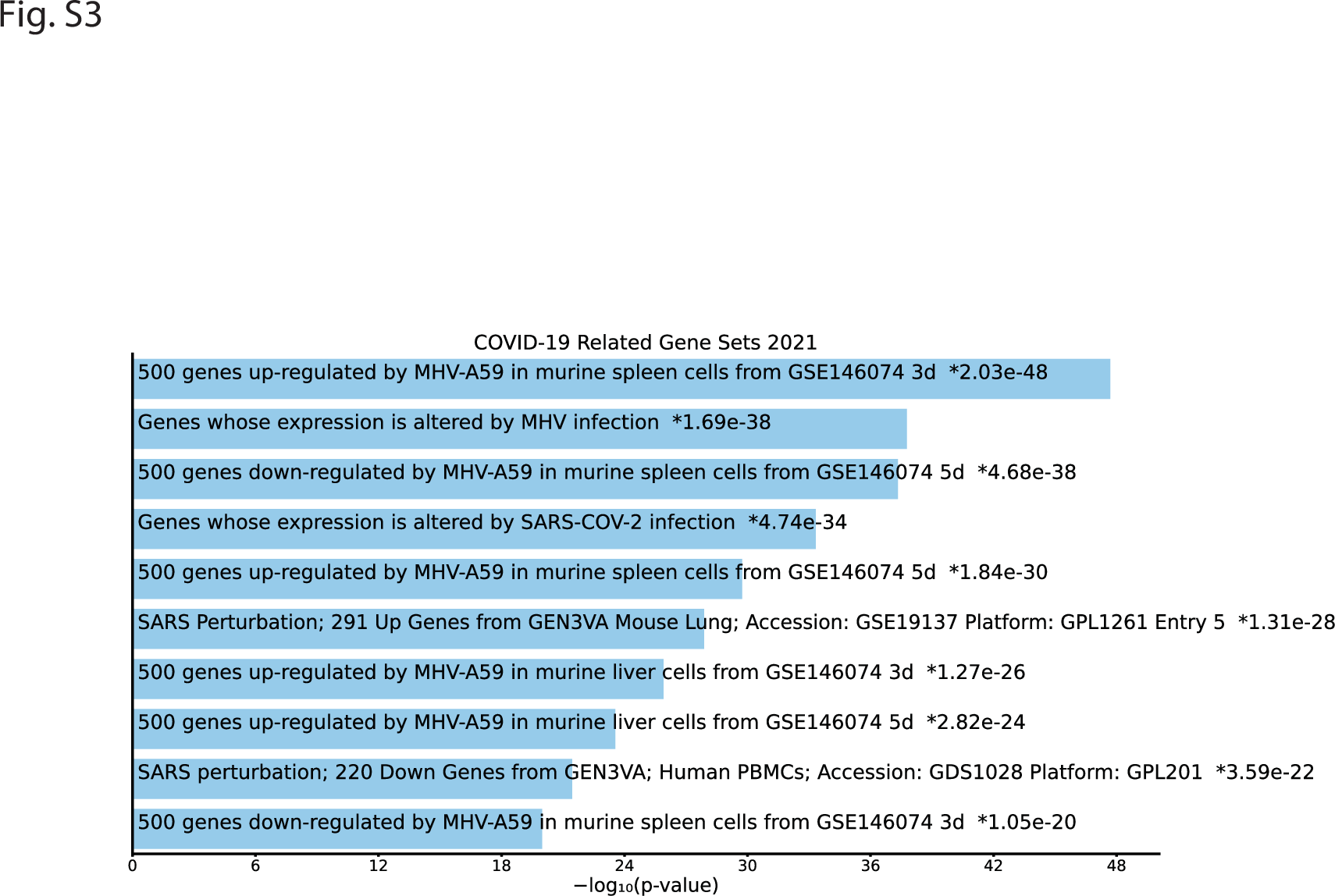

Supplement: S3 Fig — Enrichment analysis of the top 100 genes of topic 9 in the COVID-19-related gene sets. (TIF) [file pcbi.1011222.s003.tif]

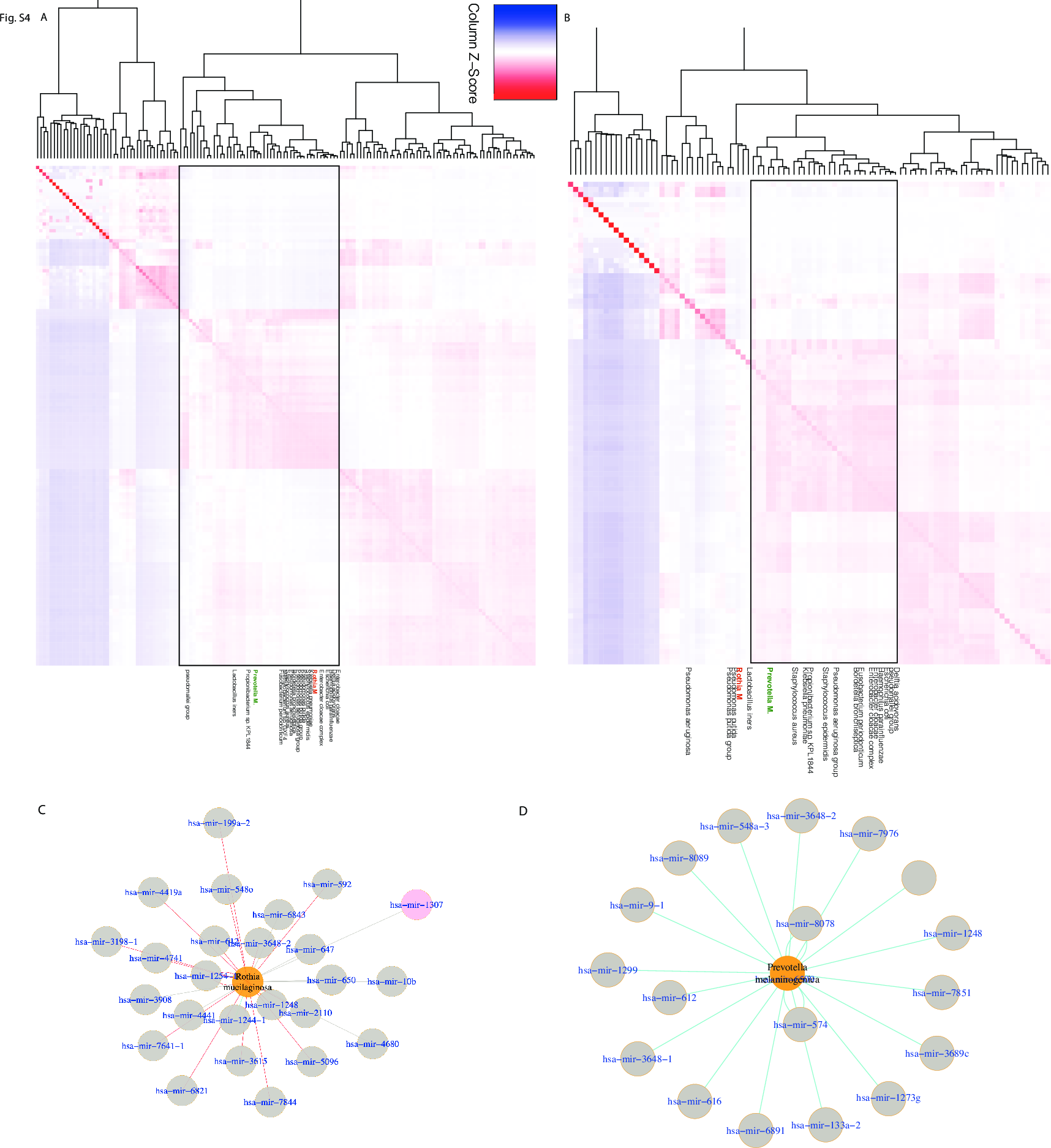

Supplement: S4 Fig — Microbe clusters in (A) COVID-19 and (B) healthy BALF tissue samples. The SARS-CoV-2-associated microbes are labeled. (C) R. mucilaginosa- and (D) P. melaninogenica-associated miRNA in COVID-19 patients. The miRNA in the pink circle is a known miRNA associated with SARS-CoV-2. (TIF) [file pcbi.1011222.s004.tif]

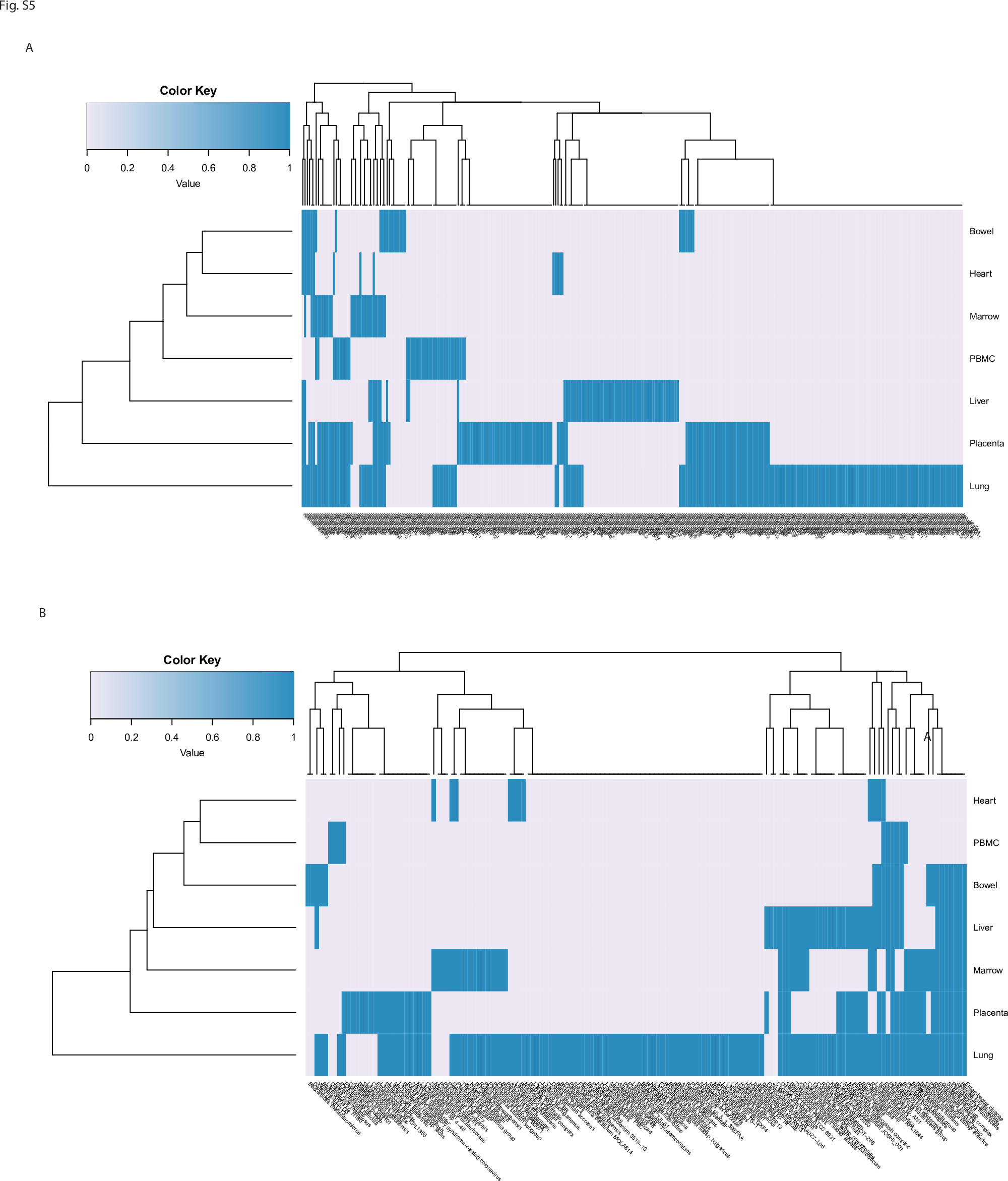

Supplement: S5 Fig — Heatmap of (A) miRNA and (B) microbe linkages across multiple tissues. (TIF) [file pcbi.1011222.s005.tif]
